# Reduced Inhibition of Return to Food Images in Obese Individuals

**DOI:** 10.1371/journal.pone.0137821

**Published:** 2015-09-16

**Authors:** Megan A. Carters, Elizabeth Rieger, Jason Bell

**Affiliations:** 1 Research School of Psychology, Australian National University, Canberra, ACT, Australia; 2 ARC Centre of Excellence in Cognition and its Disorders, School of Psychology, University of Western Australia, Perth, WA, Australia; University of Leipzig, GERMANY

## Abstract

Previous research has shown that obese individuals may be biased towards attending to food over non-food information, and this bias may contribute to the development and/or maintenance of obesity. The present study sought to extend our understanding of maladaptive attentional processing in this population by investigating whether obese individuals have difficulty in disengaging attention from food compared with non-food images, relative to normal-weight controls. To address this question, we measured inhibition of return (IOR) in an attentional cueing task. The participants were 29 obese and 35 normal-weight satiated females without eating disorders. The obese group displayed less IOR to food images than the normal-weight group, while there was no difference in IOR between the groups for non-food images. This suggests that obese females have greater difficulty disengaging attention from food than normal-weight females. Our findings provide a new focus for studies investigating maintenance factors in obesity and are discussed in relation to a theory of incentive-sensitisation.

## Introduction

In recent decades, obesity has emerged as a serious health issue of global concern [1, 2]. The adverse impact that obesity has on mental health [3, 4] and quality of life [5] has also been well-established. The emergence of obesity as a prevalent condition has been largely attributed to an obesity-promoting environment, which includes a surge in food availability that arose with industrialisation, and has resulted in continuous food cue exposure [2, 6–8].

Considering that attention is biased according to one’s current goals and motivational state [9], a vulnerability to food cue exposure in the form of an attentional bias towards food-related stimuli may contribute to the development and/or maintenance of obesity. The incentive-sensitisation theory was initially developed in the context of substance use problems [10], yet provides a potential mechanism that may underlie an attentional bias to food in obesity [7, 11]. The basic premise is that food becomes increasingly sensitised due to the dopaminergic response to food that results from repeated over-eating. The application of this theory to obesity has been supported by research showing that obesity indeed involves altered reward system functioning in relation to food [12–14]. This theoretical approach asserts that through over-eating, food stimuli gain in “incentive salience” and become increasingly attention-grabbing [7]. Detecting food rapidly and maintaining attention on food would lead an individual to be continuously reminded of its presence and availability. This may at the same time reduce the cognitive ability of that individual to attend to other, more adaptive, stimuli. It is through this process that attentional biases to food may increase the likelihood of over-eating and, in the long term, obesity.

In line with this theory, empirical research has shown an association between obesity and enhanced attentional biases to food, particularly under conditions of satiety. This was found in a study that recorded stimulus-triggered electroencephalographic brain activity, known as Event-Related Potentials (ERPs), at the P200 component, which is thought to index the intensity of attentional resource allocation in automatic information processing [15], and others recording initial eye-gaze direction to index the initial orienting of attention [16, 17]. Enhanced attentional biases to food have also been found in more sustained attention, which includes the engagement and disengagement stages of attention, and is referred to as attentional maintenance [18, 19]. Such studies include those measuring the P300 ERP component [20, 21] and eye-gaze duration [16], which are believed to reflect more conscious, maintained attention. Research has failed to consistently reproduce these associations between obesity and attentional maintenance bias to food [15, 17, 21, 22]. However, some of this discrepancy may relate to the choice of participants, with some studies including overweight and obese individuals [17, 21], and to differences in hunger across groups [15, 22].

Behaviourally, the visual-probe task has been the most commonly used measure of attention in the obesity literature. An attentional bias is characterised by faster response times to a probe that appears in the previous location of a food stimulus compared to a non-food stimulus. The visual-probe task is well-suited to examine early stages of attention through the use of short stimulus presentations (e.g., 100 or 500 ms). Researchers have also attempted to measure attentional maintenance using the visual-probe task, through lengthening the stimulus presentation duration. However, this task is limited in its ability to assess attentional maintenance as long stimulus presentations allow shifts of attention, which means it is possible that an “attentional bias” (or lack thereof) reflects the coincident direction of the eyes to one of the stimuli at the specific stimulus offset time [9, 21]. This may explain why the two visual-probe studies that have used long stimulus presentations (2000 ms) have failed to find a bias in overweight/obese individuals relative to normal-weight individuals [16, 17]. The use of behavioural tasks to accurately measure attentional maintenance biases to food is important as, once food is initially noticed, it is the maintenance of attention to food that is particularly likely to induce food cravings or prolong preoccupations regarding food. Food craving, which is considered a subjective experience of an intense desire directed at a particular food [23], may in turn trigger overeating as cravings have been shown to be related to the level of consumption of the craved foods [24]. Thus, researchers have turned to measuring inhibition of return (IOR) in attentional cueing tasks to specifically examine the disengagement of attention [25].

Attentional cueing tasks [26] involve the appearance of a central fixation cross, followed by a peripheral cue that appears in one of two possible locations, then the reappearance of the fixation cross, and finally a target either in the location previously occupied by the cue (valid trial) or the opposite location (invalid trial). The period of time between the onset of the peripheral cue and the probe onset is known as the stimulus onset asynchrony (SOA). IOR is present when there are slower reaction times on valid compared to invalid trials. This typically occurs between SOAs of approximately 300 and 3000 ms [26, 27], although the exact time period varies across studies according to factors such as sample, cue type, and task design [28, 29]. At shorter SOAs, facilitation rather than inhibition of target detection typically occurs [26]. Facilitation is represented by faster reaction times on valid compared to invalid trials, which is thought to occur due to the involuntary, reflexive attentional shift towards the source of stimulation [26]. The IOR effect is relatively robust [30], and occurs in relation to both locations and objects [31]. It demonstrates a bias against returning attention to previously-attended locations, which is thought to facilitate foraging and other search behaviours [30].

As a tool in clinical research, the IOR effect has proven useful for determining whether particular clinical groups display selective deficits in attentional disengagement. For instance, highly anxious individuals display reduced IOR to threatening stimuli, including angry faces [25, 32] and negative words [33]. Similarly, reduced IOR for negative stimuli has been found in individuals with dysphoria [34] and depression [35]. Such studies provide evidence that different forms of psychopathology involve delayed disengagement from motivationally-relevant stimuli, which may be associated with the perseverative cognitions (i.e., worry and rumination) central to these disorders.

In summary, previous research has found some evidence of an attentional bias in the processing of food stimuli in obese individuals, but there has been a paucity of research on the maintenance of attention in this context. The aim of the present study was to therefore employ an attentional cueing task to specifically examine biases in attentional disengagement from food in obese individuals. Based on incentive-sensitisation theory and evidence of attentional disengagement deficits to motivationally-relevant stimuli in other clinical groups, we hypothesised that obese individuals would display reduced IOR to food images versus non-food images in comparison to normal-weight individuals. The present study also screened for binge eating disorder (BED) to ensure that any group differences in IOR could be attributed to obesity rather than comorbid BED.

## Materials and Methods

### Participants

Flyers were placed on billboards at tertiary education campuses, health centres and local shops, and in newspapers to recruit female participants aged 18 to 63 in Canberra, Australia. A female-only sample was recruited as gender differences have been found in physiological response to food images [36], food cravings, and eating styles [37, 38]. Four criteria were used to exclude participants from the initial sample of 118. Firstly, participants were excluded if they did not have a body mass index (BMI = kg/m^2^) in the normal-weight or obese range (*n* = 16). Secondly, endorsement of a score of five or more on a scale of hunger (see below) at the time of testing led to exclusion (*n* = 1). All remaining participants had eaten a full meal within 125 minutes of their participation. This exclusion criterion was included as hunger is associated with altered attention to food [21].

The report of a binge eating frequency that was suggestive of BED constituted the third exclusion criteria (*n* = 17 and *n* = 16 for the obese and normal-weight groups, respectively). In line with the *DSM-5* diagnostic criteria for BED [39], this was determined by a response of four or more on the item of the Eating Disorders Examination-Questionnaire (EDE-Q) that measures objective binge eating, that is, “Over the past 28 days, on how many DAYS have such episodes of overeating occurred (i.e., you have eaten an unusually large amount of food and have had a sense of loss of control at the time)?” This also excluded those who responded four or more to the EDE-Q items, “Over the past 28 days, how many times have you eaten what other people would regard as an unusually large amount of food (given the circumstances)?” and “On how many of these times did you have a sense of having lost control over your eating (at the time that you were eating)?”

The final exclusion criterion was of participants who reported more than a minimal level of global eating disorder symptomatology (*n* = 4 and *n* = 15 for the obese and normal-weight groups, respectively). As suggested by previous research [40], this was based on an EDE-Q global score greater than one standard deviation above the mean of the relevant population. Norms provided by Aardoom and colleagues [41] for the general female population without an eating disorder and females with obesity without an eating disorder were used for the respective groups. Different norm sets were important as BMIs tend to be markedly elevated among individuals without eating disorders who score at or above the 90^th^ percentile of EDE-Q scores [42]. While these were the most appropriate set of norms available for the wide participant age range, they were from Belgium on a Dutch translation of the EDE-Q. Cultural differences in eating disorder symptomatology may thus have limited the applicability of these norms to an Australian sample.

The final sample consisted of 29 obese and 35 normal-weight individuals. Participants with possible depression were retained to allow for a representative sample. Additionally, as the design compared food to non-food, any cognitive slowing associated with depression was not problematic. The participants received AUS$25 or 90 minutes course credit as compensation for their participation.

### Ethics Statement

Ethical approval to conduct the study was provided by the Australian National University Human Research Ethics Committee.

### Measures

#### IOR Task

The IOR task was administered on a computer using MatLab R2012b software. It involved 48 images selected from the International Affective Picture System [43]. Images were used rather than words as they are likely to be more ecologically-valid. The images included 24 images of high-calorie food (e.g., cake, fries, roast meat, and chocolate), as research indicates that these foods particularly activate the brain’s reward system and thus may be more problematic due to greater “attention-grabbing” properties [44]. They also included 24 non-food images that were in the single category of animals (e.g., dogs, cats, and horses), as categorical homogeneity can enhance processing [45]. Each picture was 5.4 degrees wide and 3.4 degrees in height. Minor structural alterations were made to the images in standardising picture size. The sets of food and non-food images were matched at the group level and for the overall sample using independent samples t-tests (*p* > .05, two-tailed) for mean dominance, valence, and arousal ratings, as assigned by female participants in a previous study [43].

The IOR task was a spatial attentional cueing task, based on previous designs [26, 30]. Pilot testing (*N* = 8) ensured clear task demands, picture salience, and sufficient instructions, practice trials, and breaks. Participants sat approximately 60 cm from the monitor. The instructions read, “Please stare at the fixation cross in the centre of the screen at the start of each trial. When the white cross appears indicate which side of the screen it appears on by pressing the left or right arrow key. Please respond as quickly and accurately as possible. The location of the picture is not predictive of the target cross. If no cross appears, make no response and wait for the next trial.”

The task had a background colour of mid-level grey black rectangle frames were displayed 3.2 degrees left and right of the central white fixation cross, throughout the task. Each trial consisted of a 500 ms presentation of a central grey fixation cross (which remained on throughout the trial), followed by a 1000 ms appearance of a food or non-food picture in the position of one of the two black rectangles. This was followed by a change in the brightness of the central fixation cross to cue participants’ attention back to the centre of the display. Following the designated SOA for that trial, a white target cross appeared in the centre of one of the two black rectangles. The target cross thus appeared in either the cued (valid trial) or un-cued location (invalid trial). The target appeared equally on each side and equally in terms of validity status, in random order. The SOA between picture cue and target cross varied randomly between four times: 1100, 1200, 1400 and 1800 ms. Participants used the index and middle fingers on their right hand to press the left and right arrow keys, to indicate the side of the screen on which the target appeared. The following trial began after a response, or 2000 ms.

**Fig 1:**
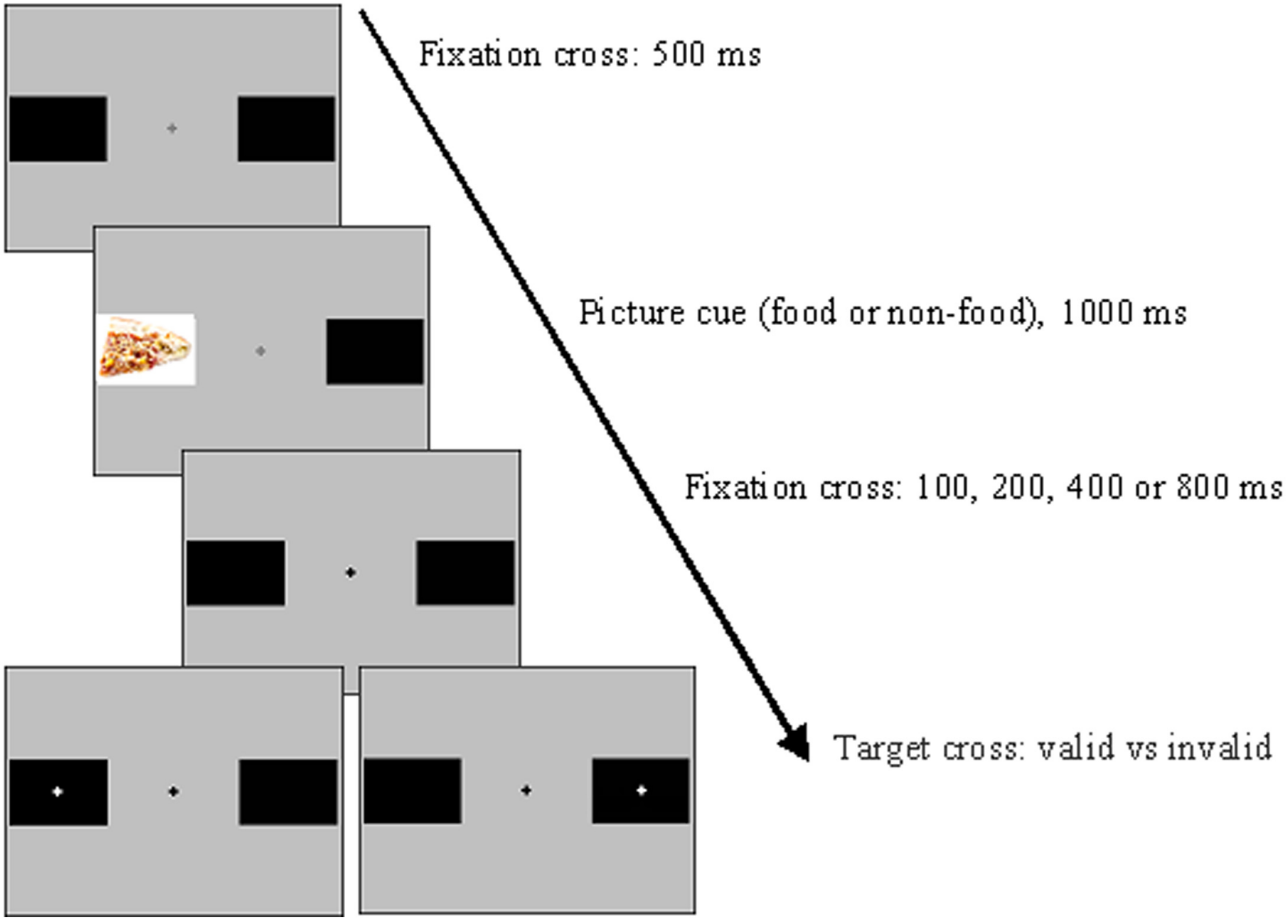
Example trial sequence.

Ten practice trials were followed by 400 true trials separated into four blocks by rest periods of a minimum of 5000 ms. In random order, the true trials consisted of 25 repetitions of each of the sixteen conditions: 2 x Picture Type, 2 x Validity State and 4 x SOA. An additional 50 trials containing no probe and thus requiring no response were included to prevent particular response rhythms. The task took approximately 25 minutes. The schematic sequence of the task is shown in Fig 1.

#### Eating Disorder Examination Questionnaire 6.0

Severity and frequency of eating disorder psychopathology was assessed using the EDE-Q [46], which is a self-report version of the interview-based Eating Disorder Examination (EDE) [47]. The EDE-Q comprises 28 items focusing on the occurrence and frequency of eating disorder symptoms over the past 28 days. 7-point Likert scales are used for the items assessing attitudinal features. The remaining items focus on specific behaviours and ask respondents to report the number of times or days that these have occurred. The items are divided into four subscales; Eating Concern, Dietary Restraint, Shape Concern, and Weight Concern. The average of these subscales provides a global measure of the severity of eating disorder psychopathology. Higher scores indicate greater severity.

The EDE-Q is widely used due to its sound psychometric properties. It has demonstrated convergent validity with the EDE [42], test-retest reliability [48, 49], and good internal consistency [41, 50]. It has also demonstrated accuracy in identifying individuals with eating disorders [41, 42] and assessing binge eating frequency [51]. Good internal consistency was evident from the Cronbach’s alpha coefficients in the present study for the Global (.88), Shape Concern (.85), and Weight Concern (.75) scales. However, the coefficients for the Eating Concern (.52) and Dietary Restraint (.60) scales were below the threshold generally considered acceptable, that is, .70 [49]. These low coefficients must be borne in mind when interpreting analyses involving these variables, although the sensitivity of Cronbach’s alpha coefficients to the number of items means it is common for them to be quite low on short scales and these scales each contained only five items [52, 53].

#### Patient Health Questionnaire-9 (PHQ-9)

The PHQ-9 [54] was used to measure the severity of symptoms of depression. It employs a 4-point Likert scale ranging from 1 (*Not at all*) to 4 (*Nearly every day*) for each of its nine self-report items. The items focus on the past two weeks and are added to form a depression severity score. While the PHQ-9 was based on the *DSM-IV-TR* criteria for major depressive disorder [55], no relevant changes appeared to this disorder in the *DSM-5* [39]. The PHQ-9 has demonstrated good construct validity [56, 57], internal consistency [58, 59] and test-retest reliability [59, 60]. Good internal consistency in the present study was indicated by a Cronbach’s alpha coefficient of .88.

#### Hunger Scale

A hunger scale was designed based on hunger measures used in previous research [16, 22]. The scale asked participants to report the time since they last ate and the degree to which they experienced hunger in that moment on a 7-point Likert scale ranging from 1 (*No hunger at all*) to 7 (*Extreme hunger*).

#### Image Emotionality Ratings Booklet

Two booklets were created by the researcher, with each containing half of the food and non-food images from the visual-probe task to reduce participant burden. In line with the ratings method employed by the IAPS developers [43], participants assigned a rating of dominance, valence, and arousal for each picture on 9-point Likert scale Self-Assessment Manikins. The scales ranged from controlled to in-control for dominance, happy to sad for valence, and excited to calm for arousal. Every second point displayed a visual representation of the relevant feeling. Item scores were summed to form dominance, valence, and arousal scores for each picture, and the ratings from the two booklets were combined.

#### Body Mass Index

Weight was measured to the closest 0.1 kilogram. Height was measured to the closest centimetre. The World Health Organization [61] BMI cut-offs were used to classify weight status as underweight (less than 18.5), normal-weight (18.5 to 24.99), overweight (25 to 29.99) and obese (30 or greater).

### Procedure

Instructions were given for participants to eat a full meal within one hour of arriving. Written consent for participation was provided. The conduct of individual testing sessions was standardised through the use of a script. They were run between 7am and 9.30pm in a private, quiet and well-lit room. The hunger scale was completed first, followed by the IOR task or another cognitive task for a separate study, in random order. A “distraction” task was completed in between the two cognitive tasks to reduce the chance of picture exposure in one task affecting responses in the other. The distraction task required participants to watch 156 seconds of music film clips taken from the Database for Emotion Analysis that had been previously rated as neutral in terms of valence, arousal, and dominance, and therefore unlikely to induce a mood effect [62]. To demonstrate that they had attended to the clips, participants responded to three questions. After completing the second cognitive task, the participants were administered (in random order) the PHQ and EDE-Q in pen and paper format. The emotionality ratings booklet was the last questionnaire administered, and height and weight were measured upon completion.

### Statistical Analyses

Data were analysed using SPSS, version 22. Screening revealed no errors of entry and no missing data. Screening for outliers revealed a relatively even split on the dichotomous variable (i.e., group), with a ratio of 1:1.2 for the obese versus the normal-weight group. Screening of the IOR task data at the individual level involved excluding reaction times less than 200 ms or more than 2.5 standard deviations above the mean, as such scores were likely due to participant disengagement and this process is typical of similar IOR reaction time studies [63, 64]. IOR Index scores were then calculated by subtracting the mean reaction time on invalid trials from the mean reaction time on valid trials. This meant that a positive IOR Index indicated a reluctance to return attention to the previously attended location, whereas a negative IOR Index indicated a facilitation effect for the valid location and the absence of IOR. The mean reaction times in each condition, that were subsequently used to calculate IOR Indices, are shown in Table 1.

**Table 1:** IOR Reaction Times for the Obese and Normal-Weight Groups for Image Type and Validity Condition (M ± SD, Milliseconds).

		Obese			
		1100	1200	1400	1800
Food	Invalid	469.24 ± 78.37	451.21 ± 84.83	444.66 ± 87.73	455.24 ± 69.87
	Valid	478.28 ± 90.12	460.28 ± 84.67	468.83 ± 85.98	476.66 ± 73.74
Non-Food	Invalid	463.31 ± 74.85	458.31 ± 82.71	451.21 ± 74.19	456.55 ± 72.65
	Valid	471.83 ± 77.12	458.76 ± 75.30	466.97 ± 91.73	474.24 ± 81.26
		Normal-Weight			
		1100	1200	1400	1800
Food	Invalid	447.17 ± 55.19	428.00 ± 55.76	420.80 ± 50.41	437.80 ± 51.41
	Valid	470.40 ± 62.51	454.06 ± 62.71	458.91 ± 62.22	467.09 ± 60.73
Non-Food	Invalid	442.31 ± 53.89	441.26 ± 61.22	440.23 ± 49.85	442.20 ± 56.24
	Valid	461.91 ± 58.34	454.14 ± 55.37	458.31 ± 59.65	456.37 ± 56.61

The results were evaluated using an alpha level of .05 and effect sizes were based on Cohen [65]. A mixed between-within subjects analysis of variance (ANOVA) was conducted to examine the research question, which asked whether the obese group would display less IOR to food images versus non-food images in comparison to the normal-weight group. The IOR scores were the dependent variables, group (obese, normal-weight) was the between-subjects factor, and picture type (food, non-food) and SOA (1100, 1200, 1400, 1800 ms) were the within-subjects factors. Analyses of the affective properties of the images were undertaken using mixed two-way between-within ANOVAs to assess whether there were differences in the food and non-food emotionality ratings, and whether any differences depended on group membership. Separate ANOVAs were conducted for dominance, valence, and arousal, with group (obese, normal-weight) as the between-subjects variable and picture type (food, non-food) as the within-subjects variable.

## Results

### Data Screening and Cleaning

Analysis of univariate outliers at the group level revealed three extreme values with standardised scores exceeding 3.29 (*p* < .001, two-tailed test). These were on the food 1100 and 1800 SOA IOR variables in the normal-weight group, and the non-food 1400 SOA IOR variable in the obese group. These cases were deemed legitimate parts of the target population and were not far beyond the cutoff (*z* = 3.50, 4.04 and 3.44, respectively), hence were retained to maximise power [66]. Analysis of multivariate outliers using the Mahalanobis Distance revealed no cases of concern (*p* < .001, two-tailed test) that were not deemed legitimate parts of the target population.

Normality was assessed at the group level using normal-weight plots, skewness and kurtosis values, and the Kolmogorov-Smirnov statistic, *p* < .05. Positive skew due to being bound by zero was present for the EDE-Q Eating and Restraint subscales. This was unsurprising, considering that eating disorder symptom measures tend to be positively skewed in the general population [67]. The non-food arousal rating and the food 1800 SOA IOR Index in the normal-weight group, and the food 1100 SOA IOR Index in the obese group also displayed positive skew. This skew is common for measures of reaction times [68]. Transformations of these EDE-Q subscales, the arousal ratings, and the IOR Indices did not improve the normality of these variables. Additionally, parametric tests are “robust” or tolerant of violations of this assumption with the sample size of the present study, thus no transformations or dichotomisations were undertaken [69]. Scatterplots for the overall sample and at the group level showed generally linear and homoscedastic relationships. Lastly, Levene’s test for equality of variances indicated that homogeneity of variance was present for all analyses where applicable, *p* > .05, unless otherwise stated.

### Preliminary Group Analyses

Group differences on the questionnaire measures were examined to understand the sample. Levene’s test for equality of variances was significant for depression, global eating disorder psychopathology, shape concern, weight concern, and BMI, *p* < .05, thus equal variances were not assumed for these variables. Independent samples t-test revealed no significant group differences for eating concern, *t*(62) = -.54, *p* = .594. Conversely, there were significant group differences in terms of depression, *t*(47.07) = -2.23, *p* = .030, global eating disorder psychopathology, *t*(43.16) = -.59, *p* < .001, dietary restraint, *t*(62) = -2.28, *p* = .026, shape concern, *t*(38.60) = -4.23, *p* < .001, and weight concern, *t*(42.06) = -7.22, *p* < .001. As expected, there was also a significant difference between the groups on BMI, *t*(62) = -14.43, *p* < .001, with the BMI range being 30 to 47.91 for the obese group and 19.49 to 24.99 for the normal-weight group. Table 2 displays the mean and standard deviation of age, BMI, depression, and EDE-Q scores across the obese and normal-weight groups.

**Table 2:** Age, BMI, Depression Scores and EDE-Q Scores of the Obese and Normal-Weight Groups.

	Obese		Normal-weight	
	*M*	*SD*	*M*	*SD*
Age	38.59	15.40	35.54	16.40
Body Mass Index	35.60	4.83	22.09	1.59
Depression	6.41	5.22	3.89	3.47
Global	1.88	.89	.92	.52
Eating	.61	.67	.53	.64
Restraint	1.58	1.1	.97	1.05
Shape	2.68	1.57	1.33	.75
Weight	2.64	1.18	.86	.66

*Note*. Depression = PHQ-9 severity score; Global = EDE-Q Global scale score; Eating = EDE-Q Eating Concern subscale score; Restraint = EDE-Q Dietary Restraint subscale score; Shape = EDE-Q Shape Concern subscale score; Weight = EDE-Q Weight Concern subscale score.

Preliminary analyses were conducted to ensure that the groups were equivalent across several variables. A chi-square test for independence revealed no significant differences in educational attainment, *χ* ^2^(6, *n* = 64) = 10.2, *p* = .117, *phi* = .40. In addition, independent samples t-tests revealed no significant differences in terms of age (obese *M* = 38.59, *SD* = 15.40; normal-weight *M* = 35.54, *SD* = 16.40), *t*(62) = -.76, *p* = .450; time since the participant last ate, *t*(62) = -1.04, *p* = .301; hunger, *t*(62) = .23, *p* = .816; and accuracy on the IOR task for the food images, non-food images and blank trials, *p* > .05. Further information on the IOR task accuracies is displayed in Table 3. The mean IOR accuracy on the catch-trials was 99.59% (*SD* = .8) for the obese group and 99.71% (*SD* = .94) for the normal-weight group. All participants achieved well above the minimum mean accuracy inclusion limit of 75%, as the minimum accuracy for any participant in any condition was 84%. This suggests the participants were engaged in and compliant with the task.

**Table 3:** IOR Task Accuracy Percentages for the Obese and Normal-weight Groups for Image Type and Validity Condition (M ± SD, Milliseconds).

		Obese			
		1100	1200	1400	1800
Food	Invalid	99.11 ± 2.03	99.11 ± 2.03	99.26 ± 1.58	98.81 ± 1.86
	Valid	98.96 ± 2.62	98.81 ± 2.68	99.41 ± 1.45	98.52 ± 2.97
Non-Food	Invalid	98.67 ± 2.94	98.52 ± 2.26	98.81 ± 2.68	99.85 ± .77
	Valid	98.81 ± 3.48	99.26 ± 1.58	98.67 ± 2.72	98.67 ± 3.51
		Normal-Weight			
		1100	1200	1400	1800
Food	Invalid	98.59 ± 2.94	99.41 ± 1.44	99.29 ± 1.55	98.71 ± 2.36
	Valid	99.06 ± 3.12	99.41 ± 1.74	99.53 ± 1.31	99.29 ± 1.84
Non-Food	Invalid	99.06 ± 1.98	99.41 ± 1.74	99.29 ± 2.08	99.41 ± 1.74
	Valid	99.18 ± 2.37	99.41 ± 1.74	99.29 ± 1.84	98.82 ± 2.52

### IOR in the Obese Versus Normal-Weight Group

**Fig 2:**
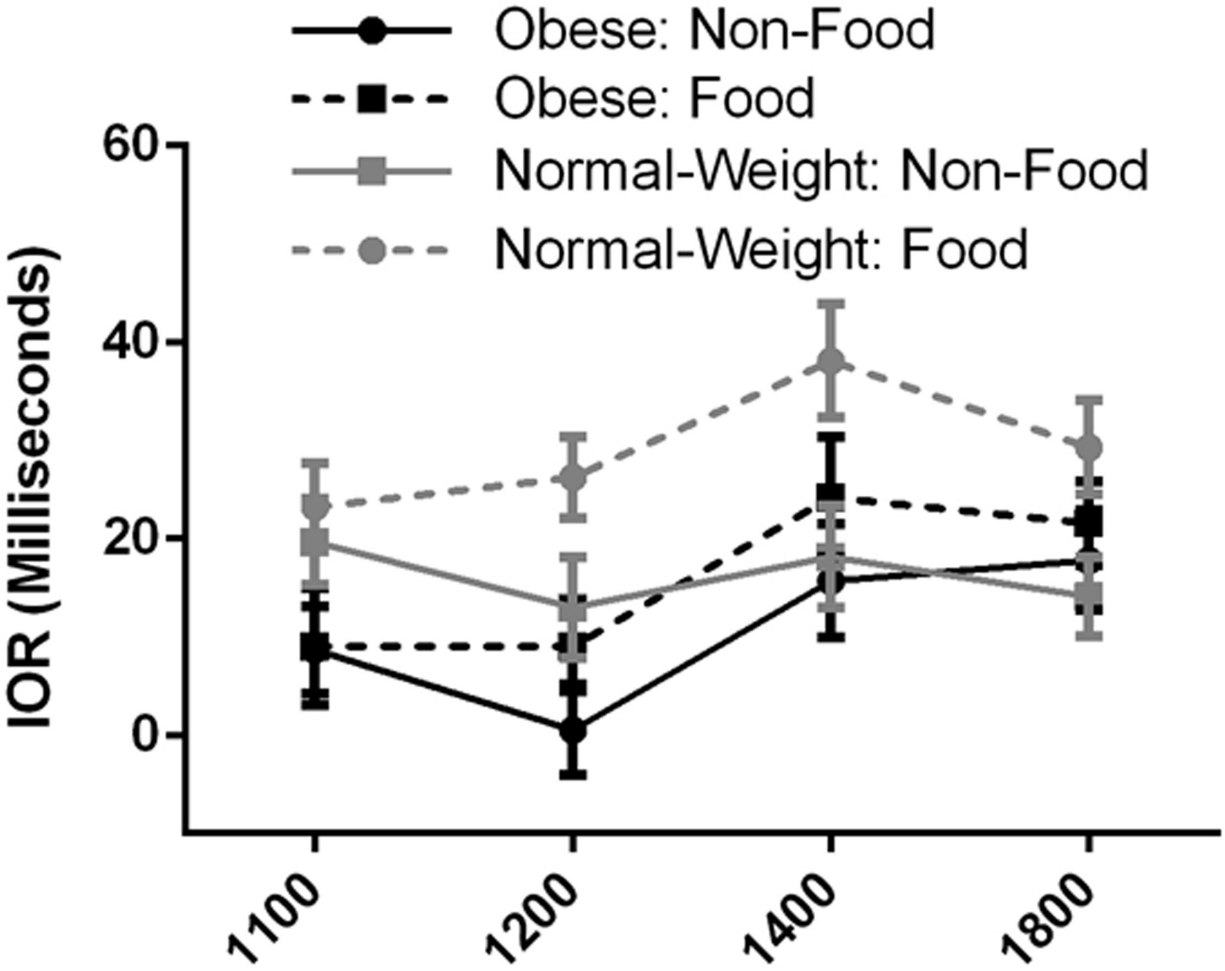
IOR Indices for the obese and normal-weight groups for food and non-food images across SOAs. Error bars: +/- one standard error of the mean.

It was hypothesised that the obese group would display less IOR to food images versus non-food images in comparison to the normal-weight group. Fig 2 shows the IOR Indices for the two groups for the two picture types across SOAs. It can be seen that some degree of IOR was present in all conditions.

Homogeneity of intercorrelations, was confirmed using Box’s Test of Equality of Covariance Matrices, *p* > .001. An ANOVA revealed a very large significant main effect for picture type, Wilks’ Lambda = .27, *F*(1, 62) = 23.41, *p* < .001, *η*_*p*_^*2*^ = .274, with the participants showing overall reduced IOR for non-food versus food images. There was also a very large significant main effect for SOA, Wilks’ Lambda = .74, *F*(3, 60) = 6.99, *p* < .001, *η*_*p*_^*2*^ = .259, with the participants showing reduced IOR at shorter SOAs, as one would expect. The main effect comparing the two groups was a moderate significant effect, *F*(1, 62) = 4.28, *p* = .043, *η*_*p*_^*2*^ = .065, with the obese group displaying less IOR overall than the normal-weight group.

**Fig 3:**
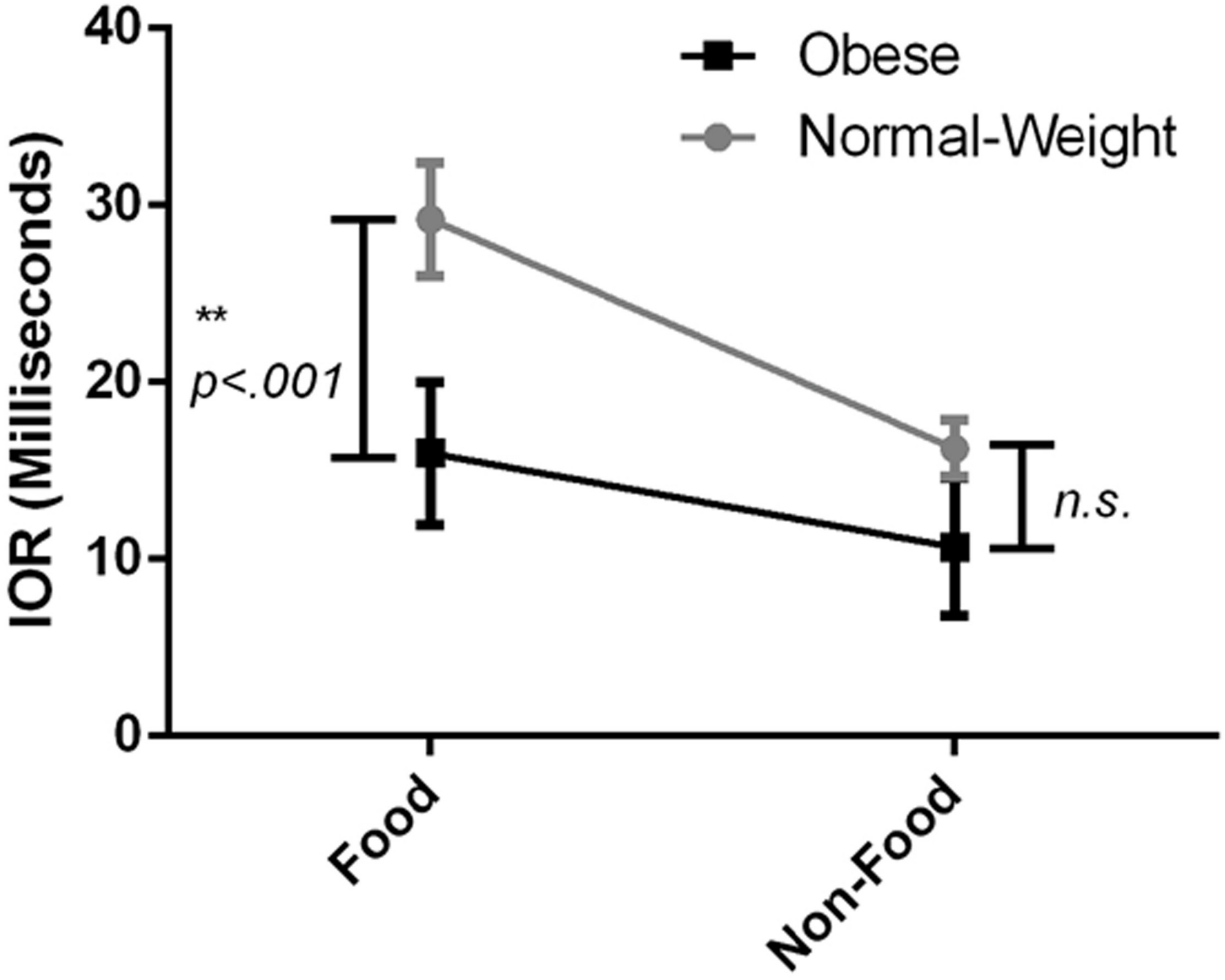
The interaction between group and picture type in predicting IOR Indices. Error bars: +/- one standard error of the mean.

However, the interpretations of the main effects needed to be qualified by the interaction effects. The results revealed no significant three-way interaction between group, picture type, and SOA, Wilks’ Lambda = .99, *F*(3, 60) = .27, *p* = .847, *η*_*p*_^*2*^ = .013. There was also no significant interaction between group and SOA, Wilks’ Lambda = .91, *F*(3, 60) = 1.92, *p* = .136, *η*_*p*_^*2*^ = .088, and picture type and SOA, Wilks’ Lambda = .91, *F*(3, 60) = 1.98, *p* = .126, *η*_*p*_^*2*^ = .09. Conversely, there was a significant interaction between group and picture type, indicating that the IORs for the two groups differed across picture type, Wilks’ Lambda = .94, *F*(3, 62) = 4.14, *p* = .046, *η*_*p*_^*2*^ = .063. Fig 3 shows the interaction between group and picture type in predicting IOR Indices.

To clarify the nature of this interaction, additional analyses were run. An two-tailed independent samples t-test revealed a significant difference between the obese group (*M* = 15.97, *SD* = 29.08) and the normal-weight group (*M* = 29.21, *SD* = 28.92) for food images, *t*(254) = 3.64, *p* < .001, with the obese group displaying less IOR than the normal-weight group. The magnitude of the differences in the means (mean difference = 13.24, 95% CI: 6.07 to 20.41) was moderate (*η* ^*2*^ = .05). Conversely, the second t-test revealed no significant difference between the obese group (*M* = 10.68, *SD* = 27.45) and the normal-weight group (*M* = 16.24, *SD* = 27.71) for non-food images, *t*(254) = 1.61, *p* = .11, with the magnitude of the differences in the means (mean difference = 5.56, 95% CI: -1.26 to 12.38) being small (*η* ^*2*^ = .01). Taken together, this analysis reveals that the obese group displayed a selective reduced IOR to food images only.

### Image Emotionality Ratings

We specifically selected food and non-food images that were matched on valence, arousal, and dominance, and these ratings were taken from normative data [43]. To our knowledge, there are no reports on whether these ratings vary in an obese population. Because of this, we decided to have participants rate our images, thus testing the prediction that their scores would match the published norms. Homogeneity of intercorrelations for the image affect analyses was confirmed with Box’s Test of Equality of Covariance Matrices, *p* > .001. Table 4 displays the emotionality ratings for the two picture types across groups.

**Table 4:** Emotionality Ratings for Food and Non-Food Images Across the Obese and Normal-Weight Groups.

	Obese		Normal-weight	
	*M*	*SD*	*M*	*SD*
Food Dominance	6.01	1.83	5.13	1.56
Non-food Dominance	5.73	1.73	4.68	1.35
Food Valence	4.52	1.11	4.40	0.96
Non-food Valence	3.27	1.32	3.66	1.02
Food Arousal	6.92	1.41	6.17	1.80
Non-food Arousal	6.06	1.75	5.93	1.63

Regarding dominance, the ANOVA revealed a moderate to large significant main effect for group, *F*(1, 62) = 6.35, *p* = .014, *η*_*p*_^*2*^ = .09. This suggested that the obese group felt more dominant over the images than the normal-weight group. There was also a moderate to large significant main effect for picture type, *F*(1, 62) = 7.64, *p* = .008, *η*_*p*_^*2*^ = .11, suggesting that the participants felt more dominant over the food than the non-food images. No significant interaction was present between group and picture type, *F*(1, 62) = .42, *p* = .518, *η*_*p*_^*2*^ = .007.

Regarding valence, the results revealed no significant main effect for group, *F*(1, 62) = .45, *p* = .507, *η*_*p*_^*2*^ = .007. This indicated that there were no group differences in the overall valence rating. However, there was a large significant main effect for picture type, *F*(1, 62) = 29.5, *p* < .001, *η*_*p*_^*2*^ = .322, suggesting that the food was rated as having more positive valence than the non-food images. No significant interaction was present between group and picture type, *F*(1, 62) = 1.92, *p* = .171, *η*_*p*_^*2*^ = .03.

Regarding arousal, no significant main effect was present for group, *F*(1, 62) = 1.37, *p* = .247, *η*_*p*_^*2*^ = .022. This indicated that there were no group differences in the overall arousal rating. Conversely, there was a large significant main effect for picture type, *F*(1, 62) = 9.95, *p* = .002, *η*_*p*_^*2*^ = .138, indicating that the food was rated as more arousing than the non-food images. However, these main effects need to be qualified by the marginally significant interaction between group and picture type, *F*(1, 62) = 3.11, *p* = .083, *η*_*p*_^*2*^ = .048, which was of moderate effect size.

**Fig 4:**
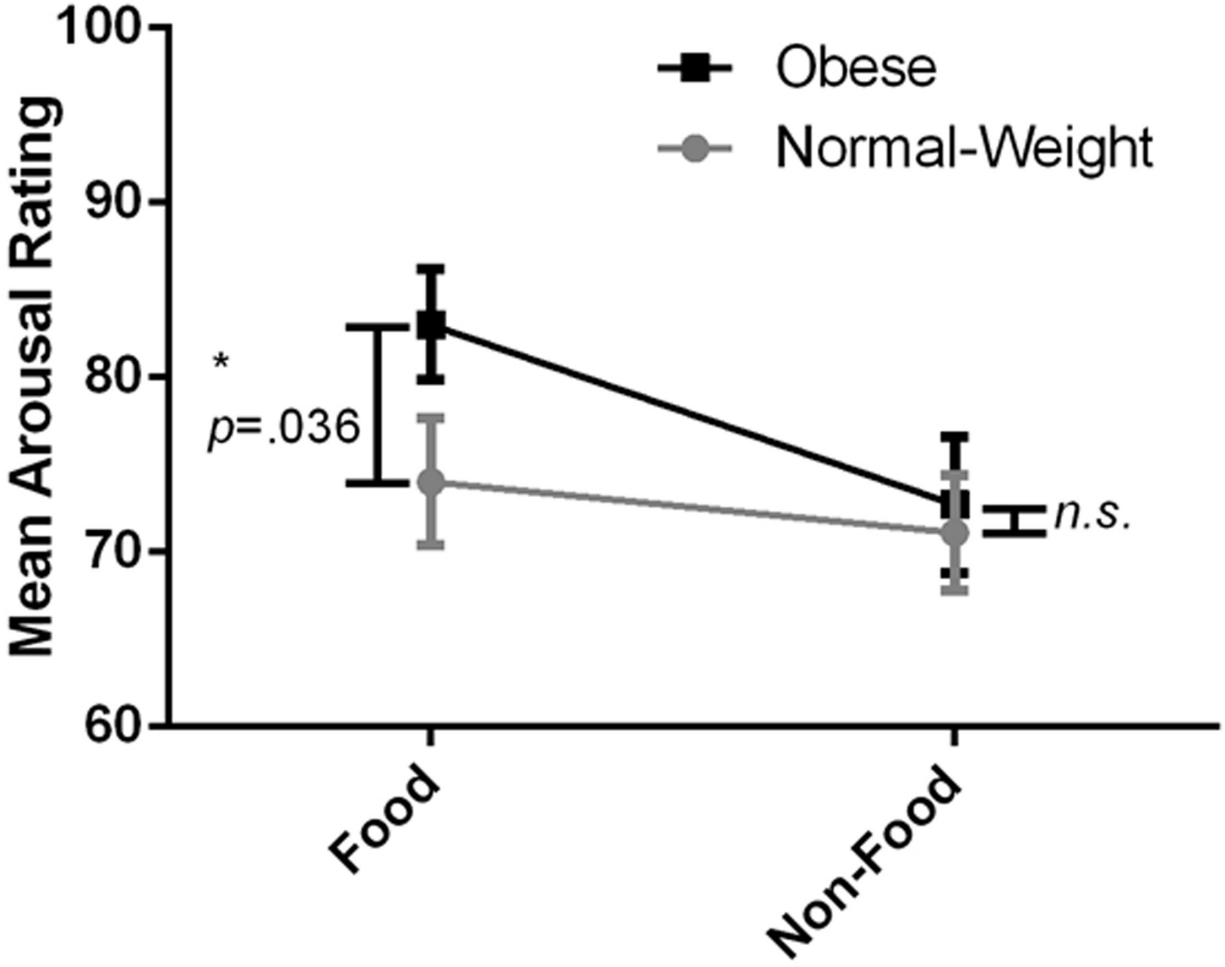
The interaction between group and picture type on arousal ratings of the food and non-food images. Error bars: +/- one standard error of the mean.

The interaction was clarified firstly by a one-tailed independent samples t-test that revealed a significant difference across groups for the food images, *t*(62) = -1.83, *p* = .036, suggesting that the obese group rated the food images as more arousing than did the normal-weight group. The magnitude of the differences in the means (mean difference = -9, 95% CI: -18.86 to .84) was moderate (*η* ^*2*^ = .051). The second t-test showed no significant difference between the obese and normal-weight groups for the non-food images, *t*(62) = -.32, *p* = .376. The magnitude of the differences in the means (mean difference = -1.61, 95% CI: -11.76 to 8.54) was very small (*η* ^*2*^ = .002). Fig 4 shows the arousal rating for the two groups for the two picture types.

Overall, the image emotionality analyses showed a difference between the groups regarding dominance ratings in general and arousal ratings for food, and between the image types for dominance, valence, and arousal. This indicates that the emotionality of images does vary between obese and normal-weight groups.

## Discussion

This study aimed to examine attentional disengagement from food images in the context of obesity. Our key finding is that the obese group displayed less IOR to food images than the normal-weight group. Importantly, this was specific to food images as there was no difference in IOR between the two groups for non-food images. This suggests that obese females have greater difficulty disengaging attention from food than normal-weight females and thus are more likely to maintain their attention on, or return their attention to, food. As anticipated in light of research on the time-course of IOR [26, 27], the IOR effect was found across all SOAs. The breadth of SOAs tested allowed for the dynamic changes in attentional processing across time to be sampled.

Previous studies have produced mixed reports regarding differences in attentional maintenance for food stimuli in obese versus normal-weight groups, with evidence for [16, 20, 21] and against [15–17, 21, 22] such differences. While previous research has focused on ERP, eye tracking or visual-probe paradigms, a notable strength of the current study is the use of a widely validated, sensitive measure of attentional maintenance, namely, the attentional cueing paradigm and resultant IOR index. The finding of a bias in the maintenance of attention to food in obese individuals when an attentional cueing task is used is contrary to the speculation that obese individuals may avoid food to manage fear of disinhibited food intake after an initial vigilance in attending to food. This was proposed by some researchers in light of the evidence for biases to food being previously less clear for attentional maintenance than for earlier stages of attention [7, 21].

These current results are consistent with incentive-sensitisation theory, which asserts that repeated over-eating produces a dopaminergic response to food that becomes increasingly sensitised [7]. In the present study, food cues indeed appeared to have increased attention-holding properties for obese individuals, which the theory argues reflects heightened food reinforcement due to increased sensitisation in the brain reward system. This finding therefore adds weight to the argument that obesity involves altered reward system functioning in relation to food [12–14], and has similarities to substance use problems in terms of the attentional processing of food and drug cues in these respective populations [10]. The central implication of the finding is that obese individuals may be continuously reminded of the presence and availability of food, easily develop desire for, or preoccupations with food, and/or have difficulties attending to other stimuli in their environment, each of which may trigger overeating. It is also possible that such a bias in attention for food cues may cause or predispose individuals to obesity.

The present study also sheds light on other factors that may have contributed to previous non-significant results regarding differences in attentional maintenance for food stimuli in obese versus normal-weight groups. Firstly, satiety in the present study was matched between the groups, whereas the normal-weight group reported greater hunger than the obese group in the studies by Nijs, Franken, and colleagues [15, 22], and participants in the study by Nijs, Muris, and colleagues [21] may not have had sufficient time to register satiety before the cognitive task. It is possible, therefore, that hunger obscured group differences in attentional maintenance. Sample differences may also help to explain the inconsistency in findings, with non-significant results possibly contributed to by the inclusion of overweight participants [17, 21] and males [15, 22], given gender differences in physiological response to food images [36], food cravings, and eating styles [37, 38].

Previous research has found general deficits in mental flexibility [70], inhibition [71, 72], and attention in obese individuals [72]. In addition to general deficits in attentional processes in obese individuals, the results of the present study suggest that there are also specific alterations in attentional processes, with the reduction in IOR greatest for motivationally-relevant stimuli, such as food.

### Clinical Implications

The present study provides a demonstration of an attentional maintenance bias in the obesity context to add to the research that has shown such biases for other clinical populations [32–35]. In doing so, this study further validates the use of attentional cueing paradigms indexing IOR for identifying and subsequently studying abnormalities in attentional processing.

The finding that obese individuals experience difficulty disengaging attention from food can inform obesity prevention and intervention initiatives in a number of ways. Firstly, public health initiatives for obesity should consider the effect of continuous and intensive food cue exposure on individuals with this difficulty. Secondly, early intervention efforts could incorporate an assessment of difficulties disengaging attention from food to identify individuals at increased risk of developing obesity. Thirdly, this finding could potentially guide current weight-loss interventions for obesity, which are currently limited, especially in the often poor maintenance of weight losses [73–75]. For instance, Attention Bias Modification (ABM) using modified visual-probe tasks has been used to reduce attentional biases to threat and symptomology in individuals with anxiety [76], and has shown promise in assisting in the treatment of depression [77] and alcohol-dependence [78]. Furthermore, initial studies have demonstrated the potential of ABM as an intervention for obesity through successfully training attention away from certain foods [79, 80], including with an obese population [81]. Interventions that aim to train attention, such as mindfulness-based therapies and Wells’ [82] Attention Training Technique, may assist individuals to specifically improve their ability to disengage their attention from food given that maintained attention is a more conscious process than the initial orienting of attention. The efficacy of such interventions in supporting obesity-prone or obese individuals to more readily disengage their attention from food should be evaluated.

### Limitations and Future Directions

The current findings need to be interpreted in light of the methodological limitations. Firstly, we did observe some differences in the perceived emotionality of the food and non-food images. Specifically, both the obese and normal-weight groups felt more dominant over the food images, and perceived these to be more positively valenced and more arousing. While the greater emotional significance of the food images for the obese group was unsurprising as it may underlie their attentional bias, this finding was unexpected for the normal-weight group as the images were matched on these dimensions of emotionality in previous research [43]. The greater emotionality assigned by the normal-weight group to the food images compared to the non-food images may have resulted in an attentional bias to food that masked a stronger difference in attentional bias between the groups. Future studies should measure the perceived emotionality of their stimuli considering that the presence and magnitude of processing biases may be mediated by differences in the emotionality of the images between their groups.

Secondly, a previous study involving an analogous attentional cueing task found that the difficulty disengaging attention from threatening images displayed by high- relative to low-anxiety individuals dissipated when threat-related response slowing was taken into account [83]. This was consistent with the notion that threat temporarily interrupts ongoing activity due to a slowing or inhibitory effect on motor responses [84, 85]. Response slowing was not measured in the present study, although unlike in this previous study [83], attempts were made to match the food and non-food stimuli in terms of emotionality. It seems unlikely that the food images in the present study were experienced as threatening; they were rated as positive in valence and, in fact, even more positively valenced than the non-food images. Furthermore, obese individuals are reported as having reduced inhibitory control, which appears inconsistent with a response slowing hypothesis [71, 72]. Nevertheless, it is an empirical question and future studies should investigate whether response slowing occurs for stimulus sets outside of the anxiety literature (e.g., food or animal images), including for those that match their stimuli sets in terms of emotionality.

Thirdly, the calorie and nutrient content of the meals consumed prior to participation was unknown. The advantages of the chosen method of controlling for hunger were that participants were allowed to eat a “normal” meal of their choice, rather than undergoing a food-deprivation period followed by the consumption of food selected by the researchers. The latter option would have carried the risk of the participants not reaching a state of satiety before the time of testing or not eating to satiety due to concerns about the amount consumed associated with obesity stigma. Low palatability of the foods provided by a researcher could also discourage participants from eating to satiety and, if they did eat to satiety, they may have experienced a “hedonic hunger” for food images [21, 86]. However, future research should continue to explore the advantages and disadvantages of different methods of controlling for hunger, and also examine whether energy intake or expenditure for the day/s preceding participation impact group differences in attention to food.

Fourth, the participants in the present study participated at a broad range of times. It would be useful for future research to examine the importance of the time of day of participation as attention to food cues may differ depending on the appeal of certain types of foods at different times of day. Future research should also investigate what effect, if any, sex-steroids have on group differences in attention to food, as these were not measured in the present study.

Lastly, the causal directions of the relationships are unknown. Preliminary research suggests that attentional bias to high-calorie foods may lead to increased consumption [87] and precede weight gain [88, 89], but limited research has been undertaken specifically on maintained attention. Longitudinal research could assist in clarifying the aetiological significance of biases in the maintenance of attention to food in obesity, such as through obtaining a sample of individuals at risk of developing obesity. Experimental research may help to establish whether induced attentional maintenance biases to food induce alterations in food cravings and eating behaviour. Finally, larger studies are required to assess whether our findings generalise to low-calorie food stimuli, overweight individuals, males, and children and adolescents.

In summary, the present study provides evidence for a selective deficit in disengaging attention from food stimuli in obese individuals without BED. This novel finding raises the intriguing possibility that increased maintenance of attention to food contributes to the development or maintenance of obesity. If this is the case, learning to more readily disengage their attention from food could help obese individuals to better cope with the current obesogenic environment of continuous and intensive exposure to food cues, which is an exciting empirical question for future research.
